# Strategies for Expanding the Operational Range of Channelrhodopsin in Optogenetic Vision

**DOI:** 10.1371/journal.pone.0081278

**Published:** 2013-11-27

**Authors:** Marion Mutter, Thomas A. Münch

**Affiliations:** Centre for Integrative Neuroscience & Bernstein Center for Computational Biology, University Tübingen, Tübingen, Germany; Institut Curie, France

## Abstract

Some hereditary diseases, such as retinitis pigmentosa, lead to blindness due to the death of photoreceptors, though the rest of the visual system might be only slightly affected. Optogenetics is a promising tool for restoring vision after retinal degeneration. In optogenetics, light-sensitive ion channels ("channelrhodopsins") are expressed in neurons so that the neurons can be activated by light. Currently existing variants of channelrhodopsin – engineered for use in neurophysiological research – do not necessarily support the goal of vision restoration optimally, due to two factors: First, the nature of the light stimulus is fundamentally different in "optogenetic vision" compared to "optogenetic neuroscience". Second, the retinal target neurons have specific properties that need to be accounted for, e.g. most retinal neurons are non-spiking. In this study, by using a computational model, we investigate properties of channelrhodopsin that might improve successful vision restoration. We pay particular attention to the operational brightness range and suggest strategies that would allow optogenetic vision over a wider intensity range than currently possible, spanning the brightest 5 orders of naturally occurring luminance. We also discuss the biophysical limitations of channelrhodopsin, and of the expressing cells, that prevent further expansion of this operational range, and we suggest design strategies for optogenetic tools which might help overcoming these limitations. Furthermore, the computational model used for this study is provided as an interactive tool for the research community.

## Introduction

The use of optogenetic tools has revolutionized neuroscience research. With the help of optical neuromodulators, it is now possible to activate or inactivate genetically targeted populations of neurons with millisecond precision, simply by shining light on the target region [1]. The general applicability of this approach has been demonstrated from worms [2] to human tissue [3]. For specific applications, certain properties are desirable for optical neuromodulators. First of all, there are two general functional classes, which either depolarize (e.g. Channelrhodopsin (ChR) [4], Volvox-Channelrhodopsin (VChR) [5]) or hyperpolarize the target cell (e.g. Halorhodopsin (NpHR) [6]). Within each class, we have optical neuromodulators that differ in their kinetic properties, in their ion selectivity (e.g. CatCH [7]) or in their wavelength sensitivity (e.g. Volvox-ChR [5]). These different functional properties arose either from the discovery of new light-sensitive proteins from different phyla, mostly prokaryotes, algae, and fungi [8] or from targeted mutations of already existing neuromodulators. The current versions of optical neuromodulators were optimized for expression level, improved transport and membrane targeting [9].

Two examples shall illustrate the breadth of ChR-variants: step function ChRs are at the extreme “slow” end of kinetic properties. Once opened by a flash of light, the channels close with a time constant of several tens of seconds [10], effectively staying open for many minutes. This can be used for opposite functional outcomes, either to increase neuronal responsiveness by elevating their baseline membrane potential [11], or to drive neurons into a depolarization block, thereby taking these neurons out of their functional network [12]. On the other end of the kinetic property range are ChETA variants like E123T or T159C/E123T [10]. Their time constants of opening and closing are in the millisecond range. As a consequence, activation by a brief light pulse will cause a single action potential in neurons, and a train of light pulses will lead to a well-defined train of action potentials.

**Figure 1:**
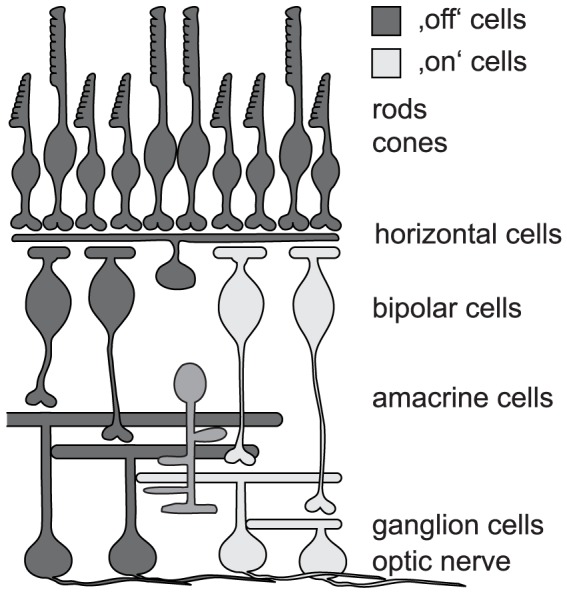
Scheme of the mammalian retina. Photoreceptors (rods and cones) hyperpolarize to light. Consequently, a successful vision restoration approach that targeted cones has utilized halorhodopsin as optogenetic tool [13]. As this strategy is using the earliest possible neurons within the retinal circuit it is most likely best suited to recreate the most meaningful (i.e. natural) light responses. There are two broad categories of bipolar cells: ‘on’ bipolar cells depolarize in response to light. They have successfully been targeted with ChR-2 to achieve vision restoration [16]. ‘Off’ bipolar cells hyperpolarize to light. Currently, there is no known promoter to drive optogene expression specifically in this cell group. Amacrine cells are a very diverse group of inhibitory interneurons. In particular A-II amacrine cells are discussed as a promising target for optogenetic intervention, as they hook into both ON and OFF circuitry in the retina. The discussion in the manuscript about targeting bipolar cells equally applies to A-II amacrine cells. Ganglion cells are the output neurons of the retina; their axons form the optic nerve. In terms of restoration of retinal processing (not just restoring light sensitivity) they are the least favored candidates for optogenetic intervention. In addition, targeting presynaptic neurons increases overall light sensitivity of the system by pooling of presynaptic input. Nevertheless, optogenetic vision restoration has successfully been performed with ganglion cells as targets [17].

Optical neuromodulators have also been suggested as a tool in prosthetic medicine [13]. One highly promising approach is their use in restoring vision after retinal degeneration [14]. In retinal degenerative diseases, such as retinitis pigmentosa, the photoreceptor cells of the retina die [15]. The lost vision can therefore, in principle, be restored by using optical neuromodulators to impart light sensitivity to neurons that are downstream of the photoreceptors, because the rest of the visual system is still intact (Fig. 1). In contrast to optogenetic applications inside the brain, the eye even provides all the optical equipment to properly guide light to the introduced optical neuromodulator. In several studies, it has been shown that ChR or NpHR can be used to make retinal neurons light sensitive, to restore retinal activity after degeneration of photoreceptors, to elicit appropriate responses in visual areas of the brain, and to activate visually guided behavior in treated animals [3], [16], [17].

The most striking difference between the native visual system, and the optogenetically restored visual function, is the lack of light adaptation. Because of adaptation, our own visual system can support vision over a range of about a dozen orders of magnitude. In contrast, with optogenetics one can activate cells only over a relatively restricted range of light intensities, spanning between 2 and 3 orders of magnitude [16]. It is important to realize, however, that also the retinal photoreceptors are restricted in their response range to 2–3 orders of magnitude at any given time, and that adaptation shifts this range depending on the ambient light level [18]. What distinguishes normal vision from optogenetic vision is therefore not so much the response properties of the individual molecular light sensors, but rather the machinery with which their responses are translated into cellular activity.

In this context, it is helpful to highlight the main difference between the way optogenetic tools are commonly used in neurophysiological experiments, and the requirements in optogenetic vision. In neurophysiological experiments, neurons are activated by an external light source which is switched on and off in an essentially binary fashion. Thus, neurons are either not optically activated (light-off condition), or they are activated by switching on the light (leading to sub- or supra-threshold activation, or even depolarization block, depending on variables like expression level or distance from the light source). In vision, on the other hand, there is always a certain ambient background light level (non-binary). The task of the visual system then basically consists of analyzing the fluctuations (also non-binary) around that ambient light level. What is more, neurons in the early visual system (photoreceptors and bipolar cells in the retina) are non-spiking neurons; their transmitter release is yet another non-binary, gradual function of their membrane voltage. Taken together, what we intend in this study, is to explore what properties of optogenetic tools might favorably support vision restoration. By taking into account the special conditions that are present in this context we highlight the possibilities of what can be achieved with ChR. We describe strategies that would double the intensity range of "optogenetic vision" compared to what is currently possible, and would therefore allow optogenetic vision in a broader environmental brightness range. Importantly, we also highlight the limitations of “optogenetic vision” that result from the biophysical properties of the channels and of the expressing cells, and we suggest that further improvements of optogenetic vision require categorically different optogenetic tools than classical ChR.

## Methods

We modeled the behavior of channelrhodopsin and cellular responses with systems of differential equations using *Mathematica* 9.0.1 (*Wolfram Research*). All details are given in the description of the results. In the Supporting Information we provide the full code (Code S1), together with an interactive user interface (Interface S1) and the description of how to use it (Manual S1). The interactive model requires either *Mathematica* Version 9 or higher, or the freely available *Wolfram CDF Player* (www.wolfram.com/cdf-player/).

## Results

In this theoretical study, our goal was to identify strategies and associated properties of ChR that would allow activation of cells over a wide range of light intensities. For the first strategies described below, we will assume that we have two variants of ChR, Variant A and B, which differ in their light sensitivity. What we mean with that is that they differ in their efficacy of depolarizing the target cell, and elicit spikes or trigger synaptic release. "Variant A" is more sensitive to light, "Variant B" less sensitive.

How can different light sensitivity, in the above sense, be achieved? The ChR-variant CatCh [7], for example, causes much stronger cell depolarization at any given light intensity than ChR-2. It is hypothesized that this effect is caused by increased permeability to calcium, which in turn triggers intracellular mechanisms that amplify the effective light response.

**Figure 2:**
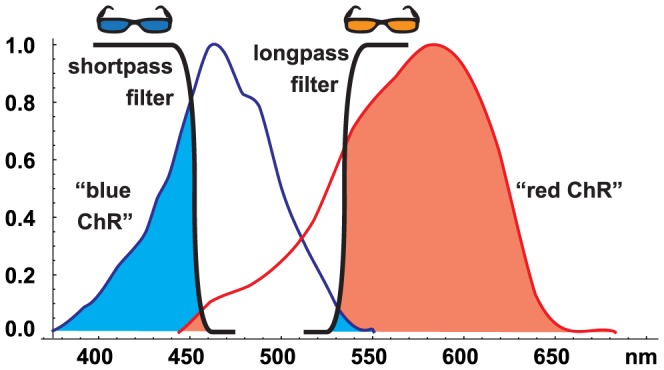
Using “colored sunglasses” to obtain ChR variants with different effective sensitivities. We take advantage of different spectral tuning of two ChR variants. One variant (“blue ChR”) has the spectral tuning of ChR-2, the other variant (“red ChR”) has a sensitivity which is shifted to longer wavelength (here illustrated with the spectral tuning curve of halorhodopsin). In this example, the cut-off wavelengths of the short- and long-pass optical filters (“colored sunglasses”) were chosen so that one variant (Variant A) has a 100 times higher apparent sensitivity than the other variant (Variant B), assuming a light stimulus with flat spectral tuning. When using the shortpass optical filter, the “blue ChR” is 100 times more sensitive than the “red ChR”; with the longpass optical filter, the situation is opposite. Note that shifting the shortpass filter to the right (or longpass filter to the left) would increase the overall apparent sensitivity of both channelrhodopsins, but the relative sensitivity difference between the two variants would become less than 100-fold.

Yet another possibility to get two versions of ChR with different light sensitivity is to take advantage of different wavelength tuning. For example, ChR-2 and derivatives of Volvox-ChR have wavelength sensitivities that overlap only in their tails [19]. If the stimulation light is restricted to wavelengths that almost completely overlap with the sensitivity range of one variant, but only with the tail of the other variant, then the two ChR variants would appear to have very different sensitivity to this light stimulus (Fig. 2). By choosing either a long-pass or a short-pass optical filter to activate this ChR-pair, either one of the two channelrhodopsins could play the role of the more sensitive ChR (Variant A) or less sensitive ChR (Variant B).

For our discussion, we do not take into account secondary effects that might increase light sensitivity, such as the intracellular mechanisms triggered by CatCh. Instead, we take as a starting point the kinetic model of wild-type ChR-2 and use this to derive desirable properties of Variant A. Our primary goal is that the sensitivity range is as broad as possible when combining Variant A and Variant B. We then discuss the necessity to also increase absolute sensitivity.

### Strategy 1: Two variants of ChR-2 in one cell

In the first strategy, we express two different ChR variants in the same cell, with the goal of broadening the effective intensity range over which that cell is responsive to light stimulation. In order for this strategy to work, there is an important requirement for the more sensitive ChR-variant: at higher intensities, those channels need to be closed. Otherwise, the cell would experience a constant depolarizing current carried by Variant A which would obscure any additional voltage modulation caused by the less sensitive “Variant B”. Thus, Variant B would not be able to modulate the cell’s spike rate or neurotransmitter output. What is needed then is that Variant A inactivates at high brightness, while having strong conductance at medium brightness levels.

**Figure 3:**
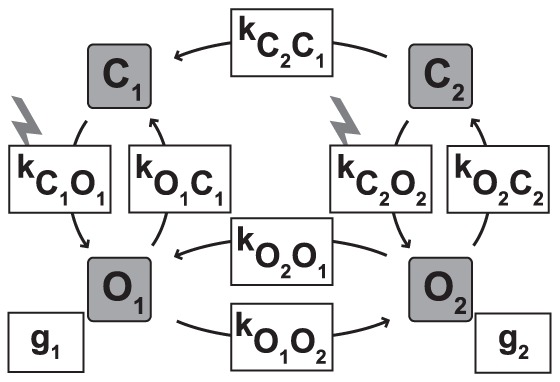
Four-state model of channelrhodopsin function. In this model, ChR has two closed (*C*_1_, *C*_2_) and two open states (*O*_1_, *O*_2_). The transitions between the states happen at certain rates; the transitions from the closed to the open states are light-dependent (indicated by the lightning). *g*_1_ and *g*_2_ are the conductances of the two open states.

Several models about the photocycle of ChR-2 have been proposed, amongst which the 4-state model (Fig. 3) has proven highly successful to fit and predict light-induced electrical currents measured in ChR-2 expressing cells [20], [21]. The 4-state model assumes two different open states of ChR-2, *O*_1_ and *O*_2_, with different conductivity, *g*_1_ and *g*_2_. It also assumes two distinct closed states, *C*_1_ and *C*_2_. Light triggers the transition of ChR-2 from *C*_1_ to *O*_1_, and from *C*_2_ to *O*_2_. In addition, the different states can thermally convert into each other with different rates. It is these rate constants that are being adjusted in our model to explore properties of ChR-2 that would better support optogenetic vision. We started with parameters that have been found to fit the responses of wild type ChR-2 [12], and then adapted the parameters to achieve appropriate properties for Variant A.

Within the framework provided by this model, we allowed ourselves to freely alter its parameters, initially without any constraints on biological feasibility. By using this approach, we can explore the maximal range of possibilities within the theoretical limits of channel biophysics. We do acknowledge that it might be very difficult to achieve certain properties by targeted point mutations, and that it might be unknown currently if a certain property might be achievable at all.

Mathematically, we represented the 4-state model as a system of differential equations, which determined the fraction of molecules that populate the four states *C*_1_, *O*_1_, *C*_2_ and *O*_2_. The transition between the states is determined by the rate constants *k* listed in Fig. 3. 
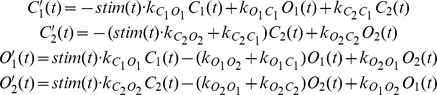



Note that in our description, for simplicity, we interchangeably use the same symbols *C*_1_, *O*_1_, *C*_2_ and *O*_2_ to describe three different things: the physical states of the channel, the probability that the corresponding state is occupied as the molecule is going through its photocycle, or the fraction of expressed ChR molecules that are in these states. Computationally, the two light-sensitive *C* → *O* transitions are implemented by scaling the respective rate constants with the stimulus intensity. In particular, we follow the convention of Hegemann et al [20] and determine the rate constant of the *C* → *O* transition by multiplying the “baseline” transition rates *k_CO_* with the light intensity, which is implemented as the time-varying stimulus *stim*(*t*). In other words, whenever there is no light, i.e. *stim*(*t*) = 0, the transition rate from *C* to *O* is 0.

We estimate the effect onto cells by the current flowing through the two open states. The current is calculated according to Ohm’s law as



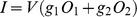
.

*g*_1_ and *g*_2_ are the conductances of the two open states and are parameters of the model, and *V* represents the cross-membrane potential, fixed at −70 mV. We use the simplified assumption here that the reversal potential of both open states is 0 mV. In the interactive model (Interface S1), the user can set the reversal potential of each open state separately.

**Figure 4:**
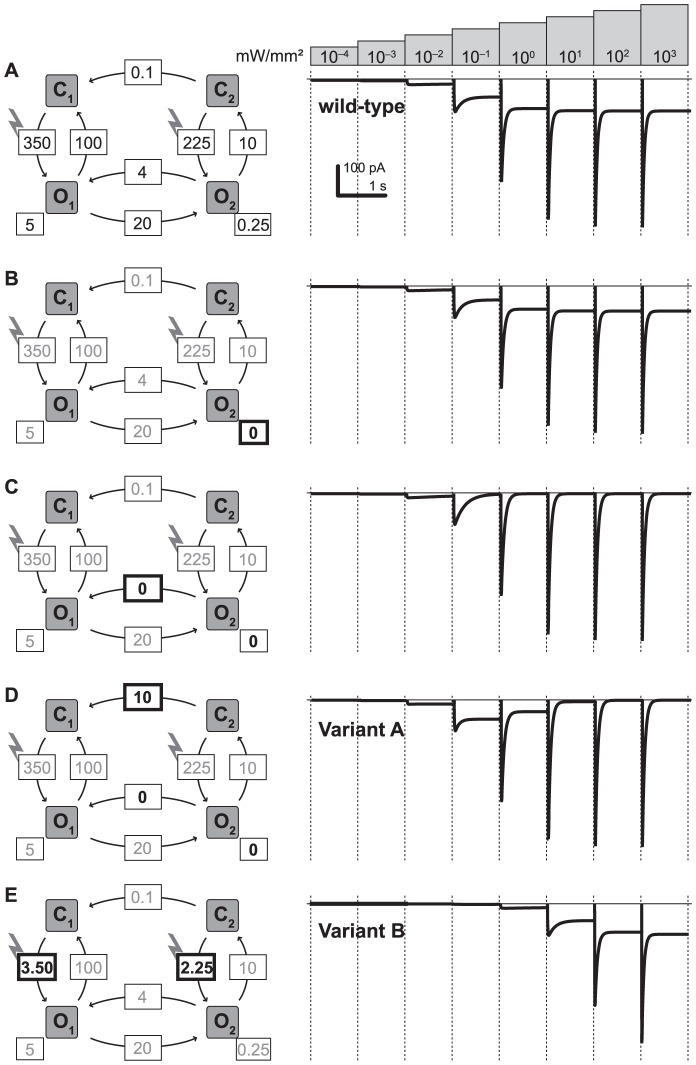
Current responses of different ChR variants to light steps of increasing intensities. In each panel, we show the light responses of a certain variant of ChR to 8 different 1-sec light steps of increasing intensity (top). At the beginning of each light step, the model was set to its basic state (*C*_1_ = 1, *O*_1_ = *C*_2_ = *O*_2_ = 0). The traces show the current flowing into the cell through the two open states, according to Ohm's law: *I* = −70 mV (*g*_1_ *O*_1_+*g*_2_ *O*_2_). **A**: wild-type ChR-2. **B-D**: By changing one parameter at a time (indicated in bold) we achieve Variant A of ChR, which has no steady state current at high intensities, while remaining such currents at medium intensities. **E**: Variant B corresponds to wild-type ChR-2 with 100-fold decreased light sensitivity.

Fig. 4 shows the cellular inward currents in response to light onset of various intensities. Before the beginning of each light step, we reset the model to the initial conditions, in which all ChR-2 molecules are in the *C*_1_ state (i.e. *C*_1_ = 1, and *O*_1_ = *C*_2_ = *O*_2_ = 0) and each light step is 10 times brighter than the previous step. Fig. 4A shows the responses of wild-type ChR-2, using the model parameters from the fit of [12]. One sees the typical behavior of ChR-2 responses to bright light intensities, in which the initial maximal current relaxes to a lower steady state. The corresponding dynamically changing occupancies of the four channel states are shown in the Supporting Information (Figs. S4 and S5 in Manual S1), and can be inspected with the interactive user interface of the model (Interface S1) also for the other light stimuli used in this study. In Figures 4B to D, we changed the model parameters one at a time. In Fig. 4B, we made the state *O*_2_ non-conductive. Despite this, the steady state current is only slightly reduced. The reason is that the states *O*_1_ and *O*_2_ are still in balance, and the steady state current is being conducted through the *O*_1_ channel state. In the next step (Fig. 4C), we prevent the transition from *O*_2_ back to *O*_1_. Consequently, the molecule gets trapped in the *C*_2_/*O*_2_ cycle: the constant light stimulus pushes the molecule from *C*_2_ back to the non-conducting *O*_2_ state, and the steady state current basically disappears. In the last step (Fig. 4D), we change the balance of the two pathways in which the molecule can escape the *C*_2_ state: We increase the rate constant for the *C*_2_ → *C*_1_ transition (alternatively, one could reduce the rate constant for the *C*_2_ → *O*_2_ transition). In weaker light, the transition now favors the direction towards *C*_1_, allowing the molecule to be activated into the conducting *O*_1_ state. At brighter light, the transition towards the non-conducting *O*_2_ state is favored. Overall, this molecule has now the properties we wish the “Variant A” ChR to have: There is no steady state current at high light intensities, while the ability to be modulated is maintained at medium light intensities.

For our strategy of combining two ChR molecules, we also need Variant B with – relatively speaking – less light sensitivity. Computationally, this can be implemented by reducing the rate constants of the light-sensitive *C* → *O* transitions. Practically, it could be achieved by Variant B having different wavelength tuning, and using the “sunglass-approach” depicted in Fig. 2. Fig. 4E depicts a possible Variant B-model. It is based on the wild-type ChR-2 (Fig. 4A) with 100-fold reduced light-sensitive rate constants. Correspondingly, the response to the step stimuli is the same as the wild-type response, albeit shifted to the right by 2 log units.

**Figure 5:**
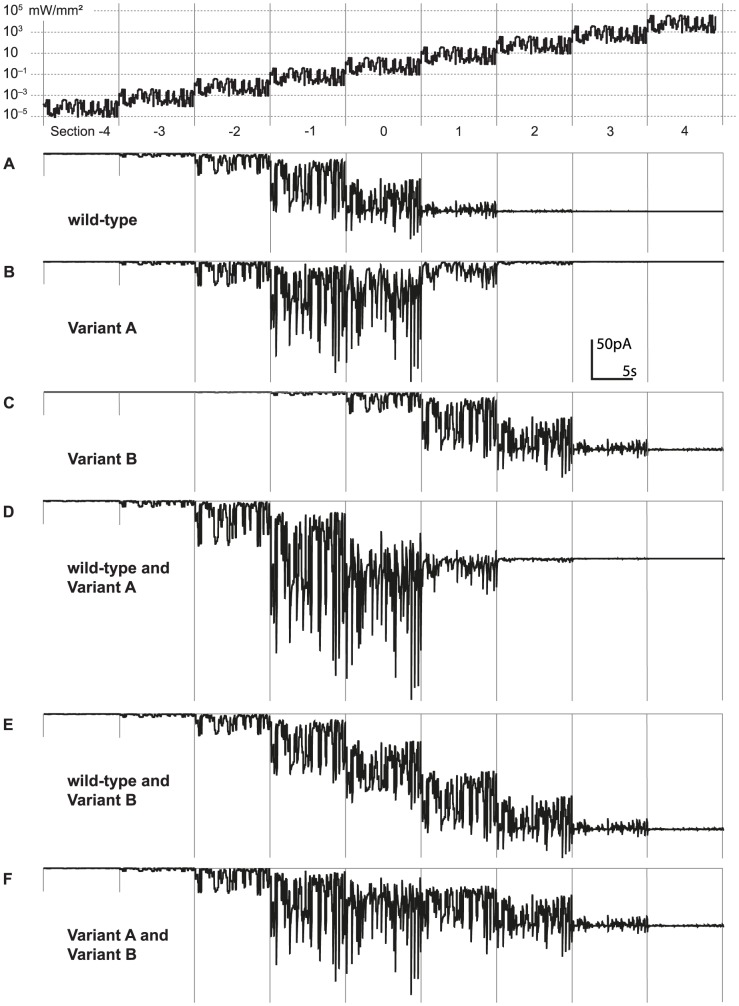
Current responses of different ChR variants to naturalistic brightness modulation at different ambient intensities. The visual stimulus (top) consisted of a naturalistic pseudo-random intensity time course spanning 2 orders of brightness magnitude. Each section of the stimulus lasted 8 seconds, then the ambient brightness level (indicated by the section number) increased by 1 log unit, and the same intensity modulation was repeated at the new level. The model was not reset at each new brightness level; the simulation of the cell's response was continuous. The traces show the current flowing into the cell through the two open states, according to Ohm's law: *I* = −70 mV (*g*_1_ *O*_1_+*g*_2_ *O*_2_). **A-C**: Current responses of cells expressing either wild-type ChR-2, Variant A, or Variant B. **D-F**: Current responses of cells expressing combinations of two ChR variants.

How do these different variants of ChR-2 fare when exposed to naturalistic light stimuli? In normal, natural vision, saccades are the dominant source of brightness changes on any point on the retina. The brightness range in natural scenes spans 2 to 3 orders of magnitude. To mimic naturalistic vision, we created a pseudo random sequence of brightness values spanning 2 orders of magnitude, changing randomly every 50 to 150 ms, i.e. corresponding to the frequency in which saccades normally occur. Every 8 seconds, we switched to a 10-fold increased brightness level and repeated the same sequence (Fig. 5, top). With this stimulus, we can evaluate the ability of the ChR-2 variants to follow naturalistic brightness modulations around different ambient levels.

The response of wild-type ChR-2, measured as inward current into the cell, is plotted in the Fig. 5A. Wild-type ChR-2 is modulated over a range of 4 orders of brightness magnitude before saturation. The responses of Variant B (Fig. 5C), by design, are identical to wild-type, shifted 2 log units to the right. Variant A (Fig. 5B), on the other hand, has distinctly different response properties. Up to section −1 of the light stimulus (the section numbers roughly correspond to mean log intensity of the stimulus), the response strength is roughly the same as for wild-type ChR-2, but then it does not keep growing. Instead, at higher intensities, responses become smaller again, until the current is completely eliminated in section 2 of the stimulus. Both wild-type and Variant A are therefore not modulated at high brightness, but while Variant A does not conduct any current, wild-type ChR-2 has a steady depolarizing effect on the cell. The combined expression of wild type and Variant A in the same cell (Fig. 5D) does not increase the intensity range over which the cell response is modulated. When Variant A and B are combined (Fig. 5F), response modulation occurs over 5-6 orders of magnitude. This result confirms the validity of our design idea. Interestingly, by simply combining two wild-type ChR-2 with different sensitivity, i.e. wild-type and Variant B (Fig. 5E), we also get modulation over the same wide brightness range as in Fig. 5F. However, the overall generated current is much larger, and might therefore drive the cell into saturation. Indeed, in our experience [16], strong expression of ChR-2 in a cell leads to saturation of the cell’s response even for a single species of expressed ChR-2 (see also Discussion).

To directly test the influence of ChR-2 activation on the membrane voltage of the expressing cell, we expanded our model and calculated the membrane potential as a function of the current flowing through ChR with the help of the membrane equation

−*CV*’(*t*) = (*V*(*t*) − *V*_rest_)/*R*+*k*_exp_ *g*_ChR_ (*V*(*t*) − *V*_reverse_)

Here, *C* and *R* are the membrane capacitance and resistance, *V*_rest_ is the resting potential of the cell (set to −55 mV), and *V*_reverse_ is the reversal potential of ChR-2 (set to 0 mV). For the membrane properties *C* and *R* we used published values for mammalian retinal bipolar cells (taken from albino rats) [22], *R* = 5 GOhm and *C* = 6 pF. *g*_ChR_ is the conductance of ChR, calculated as before as *g*_ChR_  = *g*_1_ *O*_1_+*g*_2_ *O*_2_. We included a free parameter *k*_exp_, which represents the expression strength of ChR-2 in the cell.

Given strong enough currents, the cell in our model will depolarize to the reversal potential of ChR, i.e. to 0 mV. However, a cell that is depolarized so strongly will not be able to modulate its synaptic release; it is in a state of depolarization block. For the interpretation of the following results, we will therefore assume that depolarization is useful only up to −25 mV, and that depolarization beyond this ceiling of −25 mV will not lead to more synaptic release. (Note that the envisioned target cells for our approach are non-spiking retinal neurons with a gradual relationship between membrane depolarization and synaptic transmitter release.)

**Figure 6:**
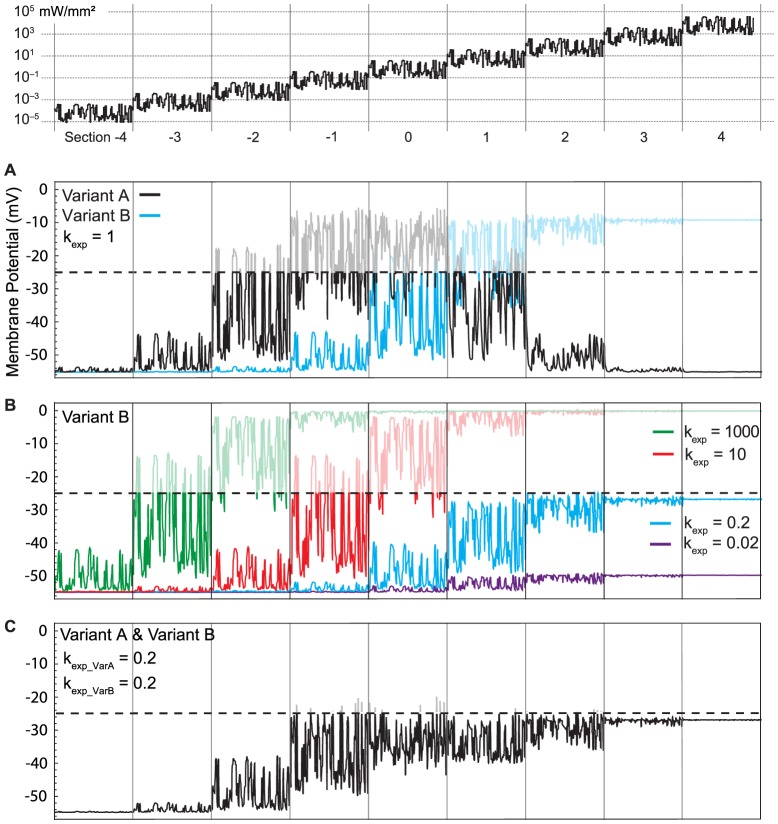
Membrane potential of cells expressing different ChR variants. The light stimulus (top) was identical to Fig. 5. The current traces from Fig. 5 are translated into membrane voltage according to the membrane equation −*C V*’(*t*) = (*V*(*t*) − *V*_rest_)/*R*+*k*_exp_ *g*_ChR_ (*V*(*t*) − *V*_reverse_) with *C* = 6 pF, *R* = 5 GOhm, *V*_rest_ = −55 mV, *V*_reverse_ = 0 mV, and *g*_ChR_ = (*g*_1_ *O*_1_+*g*_2_ *O*_2_). **A**: Membrane potential fluctuations caused by currents carried by Variant A (Fig. 5B) and Variant B (Fig. 5C). **B**: Effect of different expression levels *k*_exp_ on membrane potential modulation. **C**: Expressing Variant A and Variant B together in a single cell, at moderate expression levels, leads to modulation of membrane potential over 7 orders of magnitude. In each panel, we show the hypothetical saturation level of the cell (−25 mV). Depolarization beyond this level is indicated by dimly printed voltage traces.

Fig. 6A shows the modulation of the membrane potential of cell expressing either Variant A or Variant B, using the same stimulus as in Fig. 5. Given the high input resistance of the cell, the weak current elicited by Variant A in section −3 of the stimulus (Fig. 5B) already has a sizable effect on membrane voltage (Fig. 6A), and depolarization in section −1 can already reach and exceed our voltage ceiling of −25 mV. Due to the properties of Variant A, the membrane potential decreases in stimulus sections 1 and 2 and almost reaches the resting potential again in section 3. In contrast to Variant A, the membrane depolarization of Variant B is a monotonic function of brightness. In stimulus section 1, the cell is already fully saturated (i.e. depolarized beyond −25 mV). Note that by design, wild-type ChR would behave the same as Variant B, shifted 2 log units to darker intensity values.

The depolarizing effect on a cell depends on the expression strength of ChR in a non-linear way. The voltage traces in Fig. 6A assume an expression strength and resulting current as depicted in Figs. 4 and 5 (*k*_exp_ = 1). In Fig. 6B we investigated the effect on the membrane voltage when varying the expression strength *k*_exp_, using values of *k*_exp_ = 0.02, 0.2, 10, and 1000. Reducing *k*_exp_ to 0.2, the maximal current is reduced to a level which allows the cell to employ the full operational light-sensitivity range of Variant B without exceeding the voltage ceiling of −25 mV. This expansion of the operational range from 3 to 5 orders of magnitude came, however, at the cost of roughly 10-fold reduced light sensitivity (compare the blue traces in Fig. 6A and B). On the other hand, increasing expression rate by 10-fold and 1000-fold lead to a shift to higher light sensitivity of 1 and 3 log units, respectively. Interestingly, the increased amount of proteins (*k*_exp_ =  1, 10, 1000) influenced the modulation range only by shifting it to lower brightness levels, but did otherwise not influence the intensity-response relationship.

Combining Variant A and Variant B (Fig. 6C) at sufficiently low expression rates lead to a balanced response modulation over a wide range of light intensities, spanning 6 to 7 orders of magnitude, without ever saturating the cell. Cone photoreceptors, in natural vision, are also active only over 6 or 7 orders of magnitude. Therefore, on first sight, our approach of using Variants A and B seems highly promising. Next, we investigate the expression levels and light intensity levels that are needed to achieve the functionality depicted in Fig. 6C.

### Expression level

From the model it thus becomes clear that the expression level of ChR-2 influences the response properties of the cell (Fig. 6B). How do the numerical expression levels *k*_exp_ in our model relate to real expression levels in target cells? With *k*_exp_ = 1, the model produces a peak current of about −500 pA, given a membrane potential of −70 mV (Fig. 4A). This corresponds approximately to the currents measured in ChR-expressing neurons [1]. The single channel conductance of ChR has been reported to be as high as 1 pS, such that we would estimate that *k*_exp_ = 1 corresponds to about 7,000 channels being expressed in a cell. Other estimates for single channel conductance are as low as 40 fS [23]. This in turn would translate into about 175,000 expressed channels for *k*_exp_ = 1. However, these measurements were likely biased towards the 5 times less conductive O_2_ state, as they were performed with noise analysis under steady illumination conditions. For simplicity, we will assume a single channel conductance of about 200 fS, which translates into a number of 35,000 channels for *k*_exp_ = 1. Can we estimate an upper boundary for channels that can be expressed by a single cell? Rhodopsin molecules in the discs of the photoreceptor outer segment are packed at a density of 25,000/ m^2^ [24]. For expression levels of ChR we made the discretionary assumption of an upper bound of 1/5 of that value, i.e. 5000/ m^2^. In a cell with a membrane surface of 500 µm^2^ (rod bipolar cells [22]) we can therefore estimate that the number of expressed ChR-2 will be at most 2,500,000, corresponding to *k*_exp_ = 71. Given the approximate nature of these estimations, we will use as upper bound for the expression level a value of *k*_exp_ = 100. It has to be considered, however, that excessively high expression levels might be toxic for the target cell.

### Light intensities

**Figure 7:**
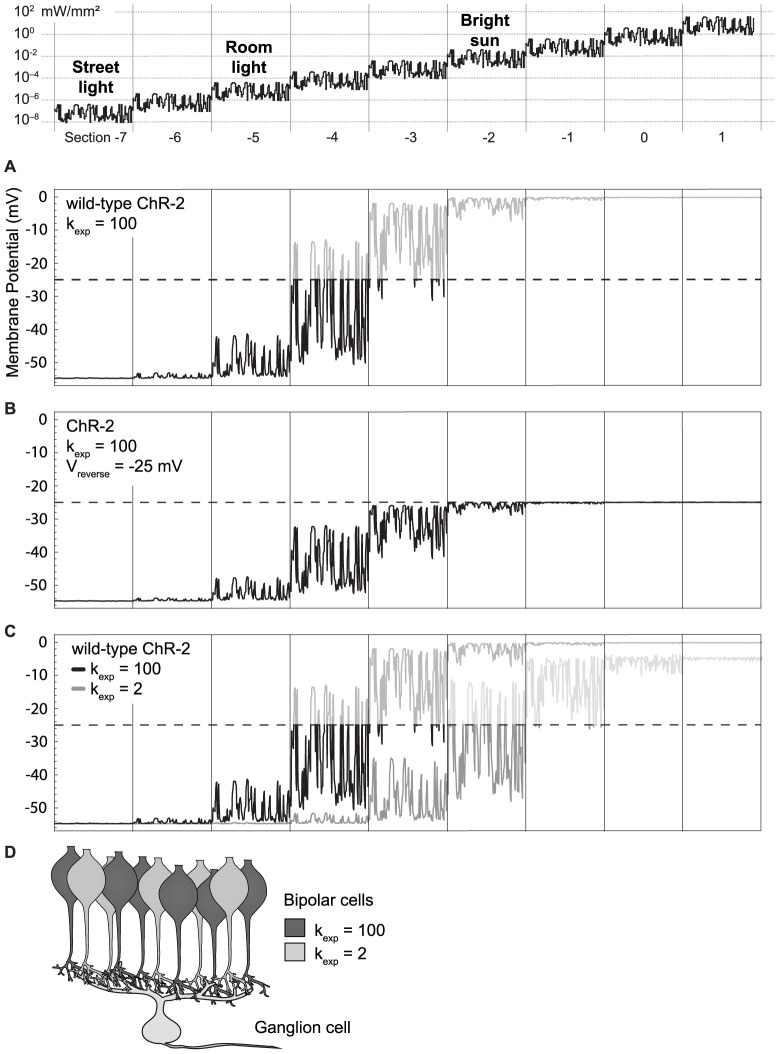
Membrane potential of cells expressing different ChR variants. The light stimulus (top) is identical to Figs. 5 and 6, with the range shifted 3 log units to dimmer intensities. Real-world luminance does not exceed 10^−1^ kW/m^2^, i.e. section −2 of the stimulus. The translation into equivalent real-world intensities is from [26]. **A**: cell response when wild-type ChR is expressed at a level of *k*_exp_ = 100. **B**: Different version of ChR with a reversal potential of −25 mV. **C**: Comparison of responses with wild-type ChR for *k*_exp_ = 100 and *k*_exp_ = 2. **D**: Schematic representation of strategy 3, expressing ChR at different levels in distinct populations of bipolar cells.

We have seen in Fig. 6B that a reduced expression level of ChR can expand the operational range, while an increased expression level increases light sensitivity at the cost of depolarization block at higher light levels. But how does the light intensity range of the model (numeric values between 10^−5^ and 10^5^) compare to the natural environment? We can estimate real light levels by the response to light steps. In the model, the peak response of wild-type ChR-2 saturated at an intensity of around 10^1^ to 10^2^ (Fig. 4A). Note that this is independent of the expression level, and a function of the biophysics of the channel itself. In real experiments, similar saturation has been achieved at 20 mW/mm^2^ when using a 470 nm (blue) LED light source [25]. Consequently, our numerical light intensity values in the model can be directly interpreted with the unit mW/mm^2^, or kW/m^2^, and we have labeled the intensity-axes in all figures accordingly. Compared to the bright blue LED light, the irradiance of sunlight, incident on the earth's atmosphere, is only approximately 1.4 kW/m^2^ (solar constant). Convolving the solar spectrum (http://rredc.nrel.gov/solar/spectra/am1.5/) with the sensitivity profile of ChR, we are left with about 10^−1^ kW/m^2^, i.e. about 1% of the value that leads to ChR saturation. Thus, bright sunlight never exceeds the intensity levels of section −2 of our model light stimulus (Fig. 7, top).

We are thus facing a conundrum: In order to increase sensitivity to a range suitable for sensing real-world light intensities we have to maximize the expression level. This, however, reduces the operational range. It would thus seem that strategy 1 is not useful as a practical strategy for optogenetic vision. Nevertheless, it is insightful to appreciate that the photoreceptors in the retina are facing the same problems, as light reception there is also based on rhodopsins. Indeed, the photoreceptors push the expression level of rhodopsin to the extreme by packing it densely into many discs in the photoreceptor outer segment. The important second step for photoreceptors is then gain control: rhodopsin activation is not directly converted into membrane voltage. Instead, a transduction cascade with many “adjustable knobs” translates rhodopsin activity into membrane potential modulation. Even this eventually reaches the limits of what is possible so that the retina is using a second trick: It employs two different classes of photoreceptors, the rods and the cones, so that each class only has to be adaptable within a more restricted range of light intensities.

In the following two strategies we will take the lessons learned from the modeling efforts leading to the impractical strategy 1, and combine them with the insights about the way the normal retina deals with broad light intensity ranges.

### Strategy 2: Adjusting the gain of ChR

Fig. 7A shows the response of a bipolar cell that expresses wild-type ChR-2 at the maximal possible level derived in the previous section, *k*_exp_ = 100. For this plot in Fig. 7, we have shifted the stimulus intensity range to the left compared to Figs. 5 and 6, in order to capture the darkest stimuli that can still activate the cell under these conditions. If, for the moment, we ignore the voltage ceiling of −25 mV, we note that the operational range of this cell spans stimulus sections −5 to −2, i.e. the brightest 4 to 5 log units present in the natural environment. Thus, luckily, at this maximally possible expression level, the full operational range of ChR-2 lies just inside the range of naturally occurring luminance. What is needed is to adjust the gain so that bright stimuli don’t drive the cell into depolarization block.

As strategy 2, we implement a simple linear adjustment of ChR gain. Such a linear adjustment can be achieved by altering the reversal potential: In our model, we assume to have a ChR-variant with a changed balance of conductivity for different ions, e.g. the channel may be more conductive for K^+^ and less conductive for Na^+^ than wild-type ChR-2. Such a variant of ChR might be achieved in the future by altering residues in the channel pore, analogous to the modifications that led to higher Ca^2+^-permeability in the variant CatCh. We further assume that this modification leads to a change in reversal potential from 0 mV to −25 mV. As a consequence, activation of the channel will never lead to depolarization exceeding −25 mV. The resulting light responses of the cell are shown in Fig. 7B. As expected, this leads to an overall dampened response, also at lower light intensities (section −5), which might be less beneficial. On the other hand, sections −3 and −2 of the stimulus are now not subject to depolarization block anymore. Overall, strategy 2 allows optogenetic vision at the four to five brightest naturally occurring log units of light intensities.

### Strategy 3: Two variants of ChR in a population of cells

Both strategies 1 and 2 relied on new variants of ChR. In strategy 3, we will exclusively build upon wild-type ChR-2. As mentioned above in strategy 2, expressing wild-type ChR-2 at the maximally possible level (*k*_exp_ = 100) will only leave room for about 2 to 3 log units of intensity range (stimulus sections −5 and −4) before driving the cell into depolarization block (Fig. 7A). When expressing ChR-2 at a lower level (*k*_exp_ = 2, Fig. 7C), activation of the cell will only start at light levels (stimulus section −3) that already produce depolarization block with high expression.

In strategy 3, we thus wish to drive expression of ChR-2 at different expression levels in *distinct* neurons of a cell population (Fig. 7D). As a result, when one half of the population is driven into depolarization block the other half is in the functional range of light intensities (Fig. 7C). This is reminiscent of the shared duties of rods and cones in the retina.

As example we will consider applying this strategy to bipolar cells in the retina (Fig. 7D). Strategy 3 would work best if the two populations of bipolar cells were distributed as homogeneously as possible. In particular, each ganglion cell at each retinal location should have presynaptic bipolar cell partners with both expression levels. This also implies that we wish to drive distinct expression levels in cells that are genetically identical (bipolar cells of the *same* type) while, at the same time, we also want to drive the same (high or low) expression level in bipolar cells of *different* genetic types. Expression level can therefore not be controlled through stronger or weaker cell-type specific promoters. Instead, a feasible strategy could be to transfect all cells of the population with ChR driven by a relatively weak promoter (leading to an expression level of about *k*_exp_ = 2, activated only at bright light), while in addition transfecting roughly half the cells with ChR driven by a stronger promoter. Transfecting only half of a cell population might in principle be achieved by a lower viral titer.

A single ganglion cell usually receives input from many bipolar cells. This spatial integration is the key to strategy 3: On the population level, the input to ganglion cells is modulated over the full 4 to 5 log unit range of light intensities, even though each of the presynaptic neurons individually only covers a 2 to 3 log unit range.

## Discussion

Normal vision can operate over a dozen log units of intensities, thanks to the adaptational abilities of photoreceptors and other retinal components. Optogenetic stimulation, on the other hand, currently only works over approximately two log units. Our computational modeling of cell responses has shown why this is the case: Even though ChR itself is modulated over a relatively wide range of light intensities (around 5 log units), the necessary high expression level in neurons reduces the effective operational range to about half of that value, as cells soon enter depolarization block (Fig. 7A). In this study, we wanted to explore possible ways how the operational brightness range of optogenetic stimulation might be expanded.

To investigate appropriate properties for ChR-2 we used the four-state kinetic model with two open states and two closed states described in detail by Hegemann et al. (2005). We provide the full Mathematica code of our model in the attached Supplementary Material. In addition, we provide a fully functional interactive user interface so that all kinetic models described in this paper can be inspected in more detail. The interactive model allows to not only look at cellular current and voltage responses, as described in the paper, but to also look at the dynamically varying occupancies of the four channel states in response to light stimulation. In addition to the rate constants and open state conductances, the interactive model also allows to freely set the reversal potential of the two open states, and the cellular membrane properties of the target neurons.

In our first strategy, starting with the properties of wild-type ChR-2, we designed a ChR-variant (Variant A, Fig. 4D) that is itself adapting: It does not conduct currents at sufficiently high ambient light intensities. Combining this Variant A with a further variant of ChR with reduced light sensitivity, Variant B (Fig. 4E), we could achieve a modulation of cell activity over 7 to 8 log units of light intensity (Fig. 6C). Unfortunately, the light levels needed to achieve such behavior are well beyond naturally occurring intensities on our planet, and thus this strategy is more of theoretical interest. One should also keep in mind that optogenetic vision restoration approaches that require two separate genes to be expressed might face difficulties to be approved for clinical trials by regulatory authorities.

In the second strategy, we took advantage of the fact that ChR-2 is itself modulated over a wider intensity range than what can be utilized in current optogenetic applications. The reason is the relatively strong gain when converting ionic currents into membrane voltage. In strategy 2, we linearly reduced this gain by setting the reversal potential of ChR in our model to −25 mV. The value chosen as depolarization ceiling, −25 mV, can be considered illustrative. The true value might be different, and it would probably be better represented as a voltage range – not a sharp boundary – over which modulations of the membrane potential are increasingly badly translated into modulations of the synaptic release. In addition, the precise value of this voltage ceiling might depend on the history of the cell’s activity. Nevertheless, it is instructive to consider such a voltage ceiling to highlight a fundamental difference between distinct applications for optogenetics. For most optogenetic applications it is necessary to very efficiently cross a threshold with brief pulses of light in order to elicit action potentials. There, it would not make sense to restrict the possible maximum depolarization – in fact, it would be highly counterproductive. In optogenetic vision restoration, on the other hand, it is helpful to prevent excessive depolarization during high ambient background illumination. Here we deal with cells, e.g. the bipolar cells in the retina, which release neurotransmitter in a gradual voltage-dependent way, and are driven into depolarization block when exceeding a certain membrane potential.

With this manipulation, cells are restricted to stay under the voltage ceiling of −25 mV, and are thus not driven into depolarization block (Fig. 7B). It is a lucky coincidence that the maximally possible expression rate of ChR in bipolar cells, derived as *k*_exp_ = 100 in a back-of-the-envelope calculation, allows us to just stay within the range of real-world light intensities. As a consequence, the brightest 4 to 5 log units of naturally occurring light intensities can be fully utilized with this approach. It should be noted, however, that the light intensities used in the model reflect the intensities arriving at the retina. Due to pupillary reduction of light intensities, the values given in the model should be increased by a factor of 2 to 20, depending on the dilation state of the pupil [26].

Finally, the third strategy is reminiscent of the rod/cone dichotomy in the retina: We create two cell populations with distinct sensitivity ranges, by expressing ChR at different levels (Fig. 7C,D). This strategy does not depend on any variants of ChR that do not yet exist. The cell population with a higher expression rate will be more light sensitive, but is driven to saturation earlier (like the rods). The other cell population is less light sensitive, and is not driven to saturation within the naturally occurring light intensities (like the cones). Whether or not such a strategy works for increasing the operational range depends on the specific biological properties of the cell pairs that are involved, namely the bipolar cells and the postsynaptic ganglion cell. The strategy would *not* work if the cells had the following two properties: The bipolar cell would constantly release neurotransmitter at a high rate when depolarized beyond −25 mV (i.e., there would be no modulation of release, but also *not* a depolarization block in the bipolar cells), and the ganglion cell would be driven to saturation by this steady state release of half its presynaptic bipolar cells (i.e. there *would* be a depolarization block in the ganglion cell). If any of the two conditions is not met, strategy 3 could be viable.

Strategies 2 and 3 require very high expression levels to achieve the desired goals of expanding the operational range of optogenetic vision. This requirement might be alleviated by using variants of ChR that have higher light sensitivity, for example the variant CatCh discussed in the introduction [7], or the tandem protein vChR1-ChR2 [27], which is sensitive over a broader wavelength range and can thus effectively utilize more of the incoming photons. As CatCh probably achieves this higher sensitivity by increased conductivity for calcium ions, it might be difficult to make it compatible with strategy 2, which depended on changing the reversal potential of ChR. Strategy 3, on the other hand, might benefit from variants like CatCh, as one can either shift the sensitivity to dimmer ranges, or achieve the same sensitivity with less expression.

All strategies we presented here exclusively worked by tweaking the biophysical properties of the channel, or by manipulating the expression level. Although these strategies promise to increase the operational range roughly two-fold to cover the brightest 5 log units of naturally occurring light intensities, we are still far away from the operational range of natural vision. Our results suggest that further expansion of the operational range is not possible with channelrhodopsins, due to the limitations of the biophysics of the channel itself, and of the expressing cells. Optogenetic vision with channelrodopsins at the most relevant brightness ranges, i.e. at mesopic intensities, would thus require technical tools like image intensifiers. Pure optogenetic biological solutions that cover a much wider range of intensities will likely require future developments of optogenetic tools that link the optical neuromodulator to intracellular signaling cascades that enable adjustment of the response gain. First strides in this direction have already been undertaken [28], [29]

## Supporting Information

Code S1 Mathematica Code of the interactive model as text document. The code can be copied and pasted into *Wolfram Mathematica* and executed there. It requires *Mathematica* version 9 or higher to run.
(TXT)

Interface S1 Standalone interactive user interface of the computational model as a *Wolfram CDF* document. This interface can be opened either in *Wolfram Mathematica*, or in the freely available *Wolfram CDF Player* (www.wolfram.com/cdf-player). Version 9 or higher are required to run the interface.
(CDF)

Manual S1 Explanation of how to use the interactive model of channelrhodopsin light responses (Interface S1). This document also contains the Figures S1 to S5.
(PDF)
