# Supplementary material for: Strategies for Expanding the Operational Range of Channelrhodopsin in Optogenetic Vision
Source: PLoS One. 2013 Nov 27;8(11):e81278. doi: 10.1371/journal.pone.0081278 (PMC3842264; doi:10.1371/journal.pone.0081278)
Supplement: Manual S1 — Explanation of how to use the interactive model of channelrhodopsin light responses (Interface S1). This document also contains the Figures S1 to S5. (PDF) [file pone.0081278.s003.pdf]

Supporting Information for

Strategies for expanding the operational range of channelrhodopsin in optogenetic vision
Marion Mutter & Thomas A. Münch

We supply the full code of the computational model as Supporting Information. The model was written in *Mathematica* 9.0.1 (*Wolfram Research*), and it requires at least version 9 of *Mathematica* to be executable. With *Mathematica* 9 or later, the model can be executed, and the code can be freely adjusted and changed by the user.

The interactive user interface can also be explored with the *Wolfram CDF Player* (at least version 9), which can be downloaded for free from the *Wolfram* Website (www.wolfram.com). With the CDF player, no adjustments to the code are possible, but all parameters of the model can be changed interactively as described in this document.

The supporting information consists of three files:

1) **Manual S1** (this document, size: 728 kB)
   Description of the model's user interface, including Figures S1 to S5

2) **Code S1** (Size: 28 kB)
   The Mathematica code of the model as plain *text* document. The code can be copied-and-pasted into *Mathematica*. There, it can be executed to generate the user interface.

3) **Interface S1** (Size: 52 kB)
   Just the interactive user interface as *Wolfram Computational Document Format* (CDF) file.

Description of the interactive user interface

I. Overall user interface

The user interface is divided into 3 panels (Fig. S1):

Panel 1: Interactive panel to set the rate constants of transitions between the channelrhodopsin states C_1, C_2, O_1 and O_2; the conductances g_1 and g_2 of the two open states; and the reversal potentials $V_{rev}O_1$ and $V_{rev}O_2$. The three buttons on top allow setting the model parameters according to three ChR-Variants discussed in the paper (WT, Variant A, Variant B).

Panel 2: Here, the user can save three arbitrary versions of ChR. The button >>> saves the present setting of panel 1; a thumbnail image of the current model will be displayed in panel 2. Pressing the button <<< writes the saved settings back to the interactive panel 1.

Panel 3 shows the responses to light stimuli.

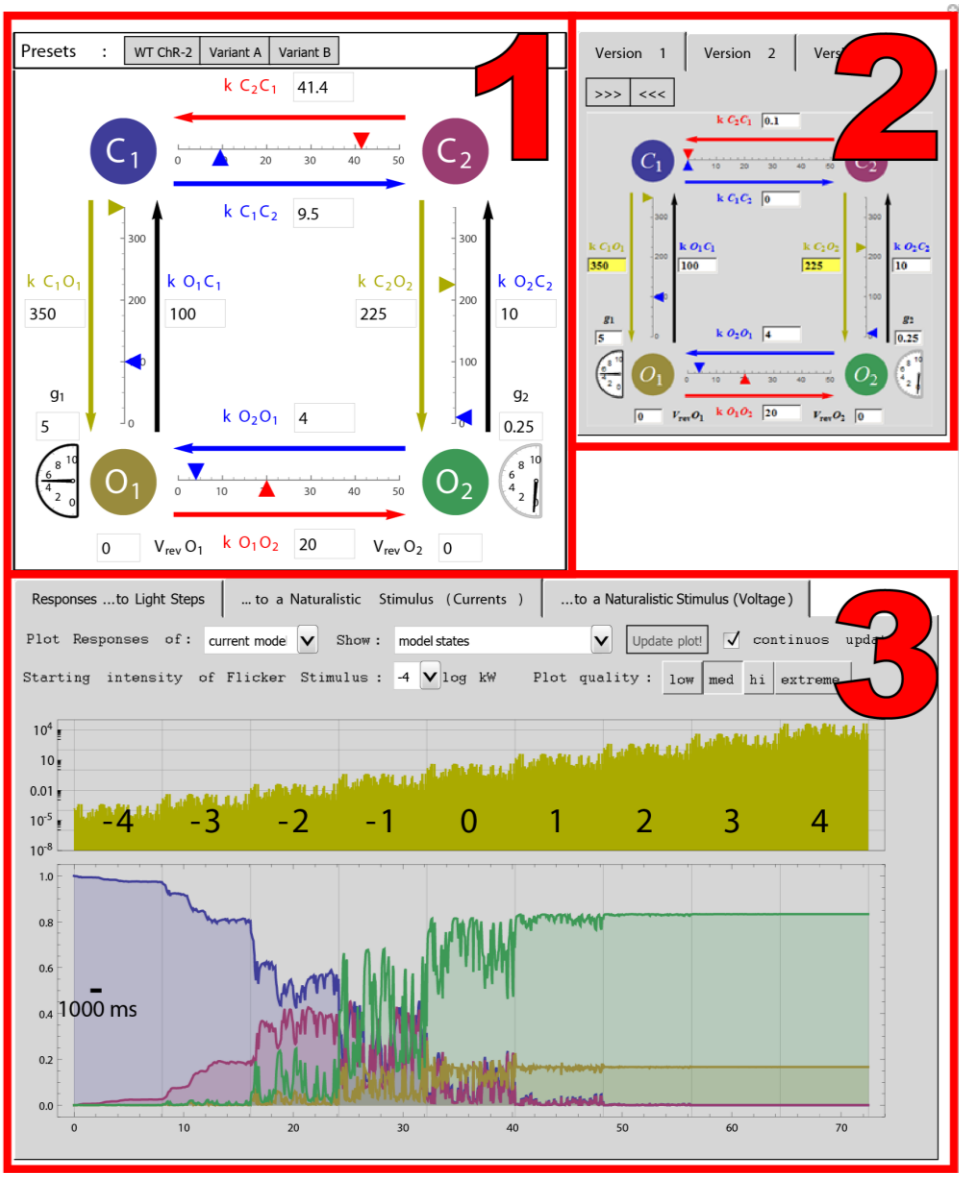

Fig. S1 User interface of the interactive model *The user interface consists of three panels.*

II. Setting the model parameters

The following model parameters can be set interactively in panel 1 (see Fig. S2):

1. Setting the rate constants:
The rate constants can be set by moving the sliders (Δ) with the mouse, or by typing the values into the text fields. While the range of the sliders is limited (e.g. 50), arbitrary numeric values can be entered (e.g. 70, see example highlighted by the red ellipse). Holding down the ALT-key while moving the sliders allows fine adjustments of the settings.

2. Setting the conductances of the open states:
The conductances can be set by moving the gauge with the mouse, or by entering a numeric value into the text field. While the range of the gauge is limited, arbitrary numeric values can be entered. Holding down the ALT-key while moving the gauge allows fine adjustments of the settings.

3. Setting the reversal potential of the open states:
The reversal potentials are set by entering numeric values in the text fields.

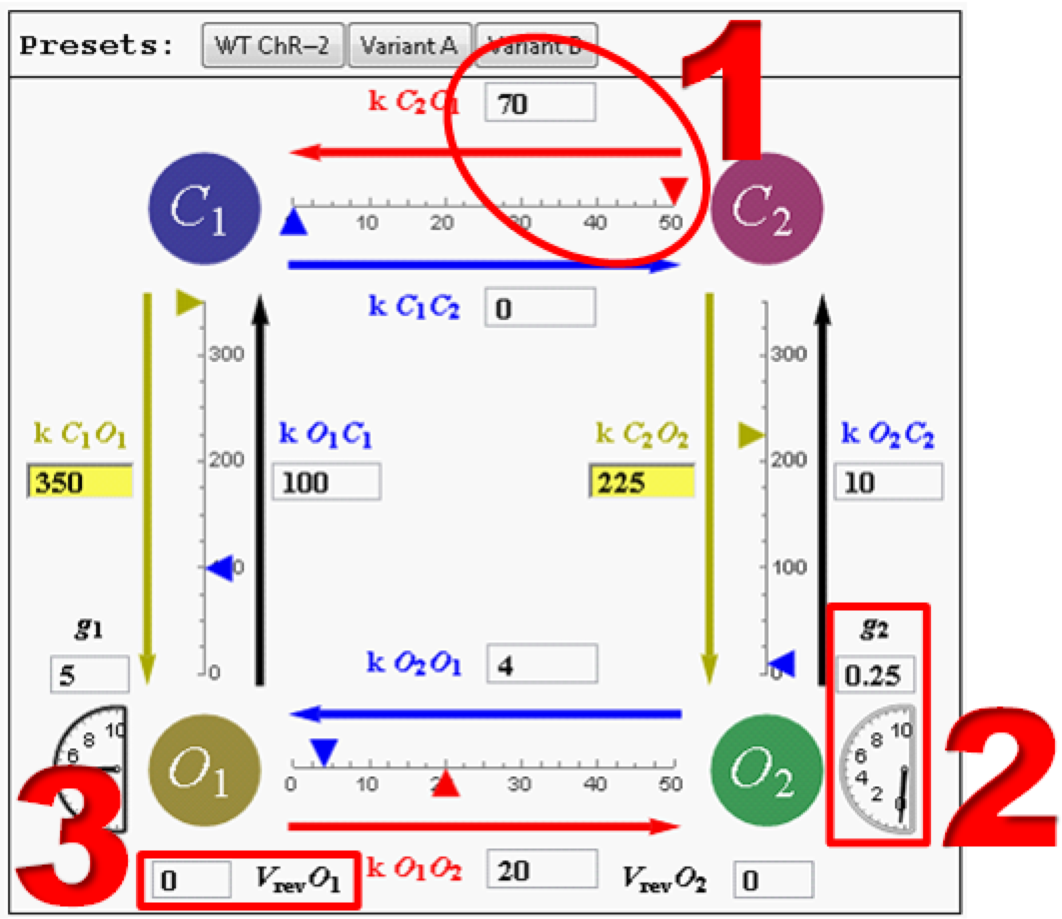

Fig. S2 User interface of the interactive model
The first panel of the interface allows setting the parameters of the model.

III. Plotting light responses (Fig. S3)

Light responses can be plotted in response to step stimuli (first tab, Fig. S3 A), or to a naturalistic stimulus (second and third tabs, Figs. S3 B and C). In all three tabs, the plots can be set to continuously update when parameters are changed. If the corresponding checkbox is not checked, the button [Update plot!] needs to be pressed. More details about the individual sub-panels are given below.

a. Responses to light steps

When showing the responses to light steps, the user can chose between 7 different versions of ChR (see Fig. S3 A_1). "Current model" refers to the present settings in Panel 1. "WT", "Variant A" and "Variant B" refer to the three variants discussed in the paper. "Version 1" to "Version 3" refer to the three variants saved by the user in Panel 2. The user can pick to either show the current flow through the open states of the ChR model, assuming a clamped membrane potential of V_m = –70 mV, or the occupancies of the four channel states (see Fig. S3 A_2). In the plots of the occupancies of the four channel states, the four states are color coded according to the colors in Panel 1 (e.g., blue corresponds to state C_1, purple to state C_2, etc., see Fig. S1).

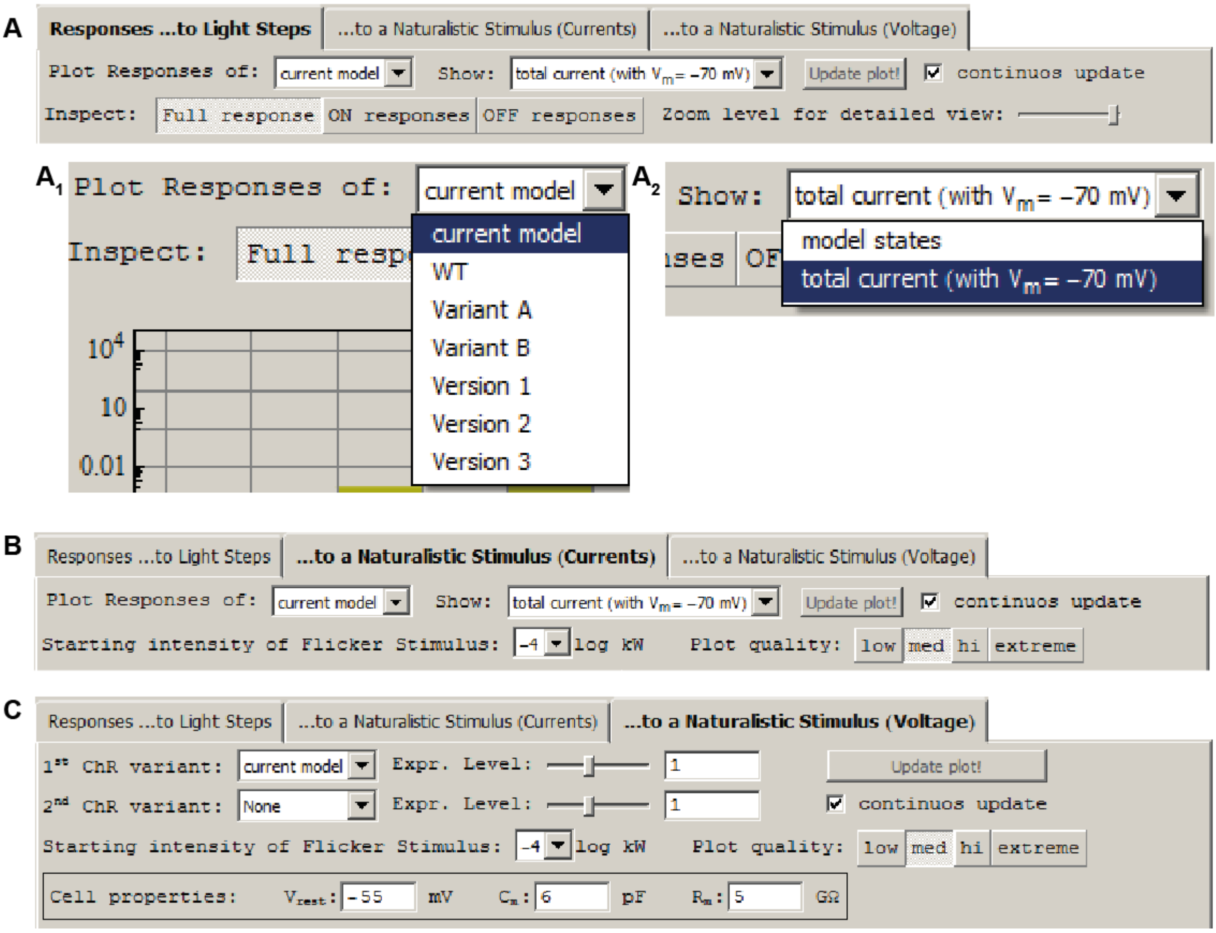

Fig. S3 Controls for showing responses to different light stimuli
A, Controls for showing responses to light steps. B, controls for showing current responses to naturalistic stimuli. C, Controls for showing voltage responses to naturalistic stimuli.

Fig. S4 shows the channel states of WT ChR-2 in response to the step stimulus. Note that the model is being reset to the initial state (i.e., 100% of the channels are in state C_1) before the onset of each step stimulus. In addition to showing the full response, the user can more closely inspect the responses to the onset or the offset of the stimuli, by selecting the appropriate button above the plot. Zooming into the response can be achieved with the slider on the right. Fig. S5 shows the same responses as Fig. S4, with an expanded time scale and focus on the ON and OFF responses, respectively.

b. Current responses to a naturalistic stimulus

When showing the responses to a naturalistic light stimulus (Fig. S3 B), the user can make the same choices as for the step stimulus (One of seven different ChR variants, showing currents or model states). In addition, the user can choose to let the stimulus start at different intensities. On slow computers, it may be advisable to reduce the plotting quality. Lower plotting quality will lead to less faithful representation of fast transients in the responses.

c. Voltage responses to a naturalistic stimulus

When plotting the voltage responses of the cell expressing ChR, the user has additional free parameters. First of all, the membrane properties of the expressing cell can be set by entering the appropriate values into the input fields (Fig. S3 C). In addition, the user can choose to express up to two variants of ChR in the cell. For each variant, the expression level (called k_{exp} in the paper) can be adjusted by moving the corresponding slider, or by entering a value into the text field (Fig. S3 C).

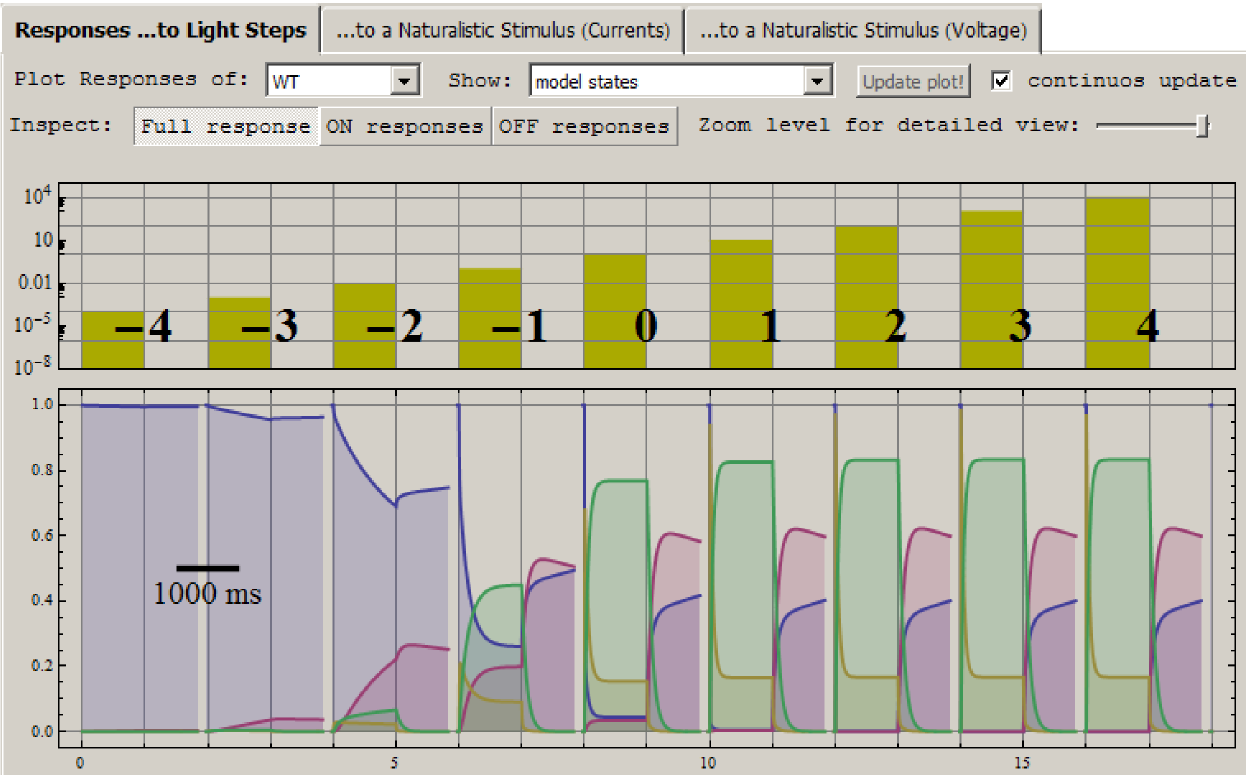

Fig. S4 Step responses of WT ChR-2
Channel occupancies of WT ChR-2 in response to square steps of light of increasing intensity. At the beginning of each step, the model is reset so that 100% of the channels are in the C_1 state. The four lines in the plot are color-coded according to the interactive model diagram in Panel 1 (see Figs. S1 and S2).

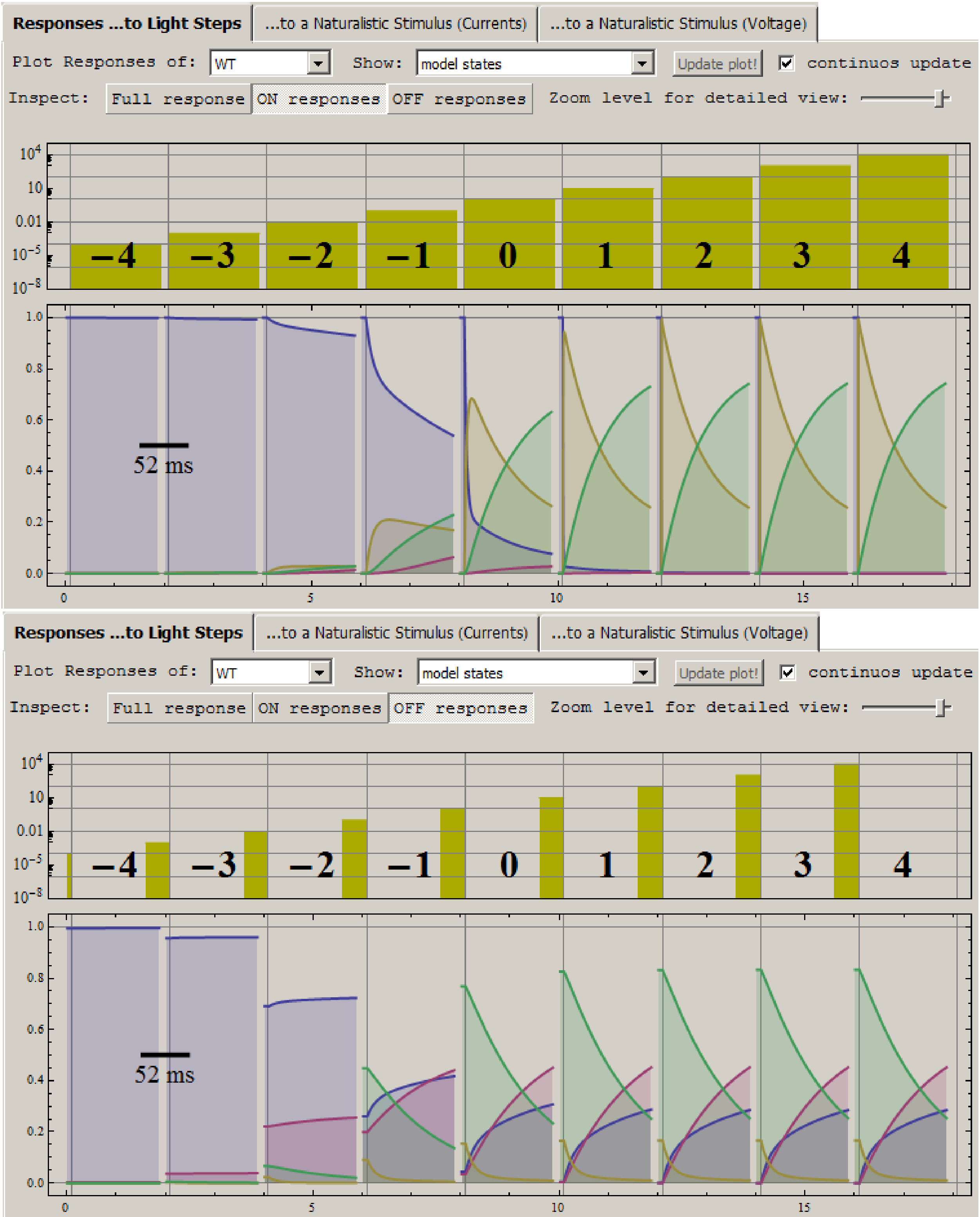

Fig. S5 Step responses of WT ChR-2 – detailed view

Same responses as in Fig. S4, with expanded time scale and focus on the ON response (top) or OFF response (bottom), respectively. The zoom level can be adjusted with the slider above the plot.
